# Supplementary material for: Sperm modulate uterine immune parameters relevant to embryo implantation and reproductive success in mice
Source: Commun Biol. 2021 May 14;4:572. doi: 10.1038/s42003-021-02038-9 (PMC8121928; doi:10.1038/s42003-021-02038-9)
Supplement: Supplementary file 1 — Supplementary Information [file 42003_2021_2038_MOESM1_ESM.pdf]

Supplementary Information for

Sperm modulate uterine immune parameters relevant to embryo implantation and reproductive success

Authors: John E. Schjenken*, David J. Sharkey*, Ella S. Green*, Ricky A. Matias*, Lachlan M. Moldenhauer*, and Sarah A. Robertson*‡

Author affiliations: *The Robinson Research Institute and Adelaide Medical School, University of Adelaide, Adelaide, SA 5005, Australia

‡**Corresponding author:** Sarah A. Robertson, The Robinson Research Institute, Adelaide Medical School, University of Adelaide, Adelaide, SA 5005, Australia.

Phone: +61883134094

E-mail: sarah.robertson@adelaide.edu.au

This PDF file includes:

Supplementary Figures 1 to 9
Supplementary Tables 1 to 3

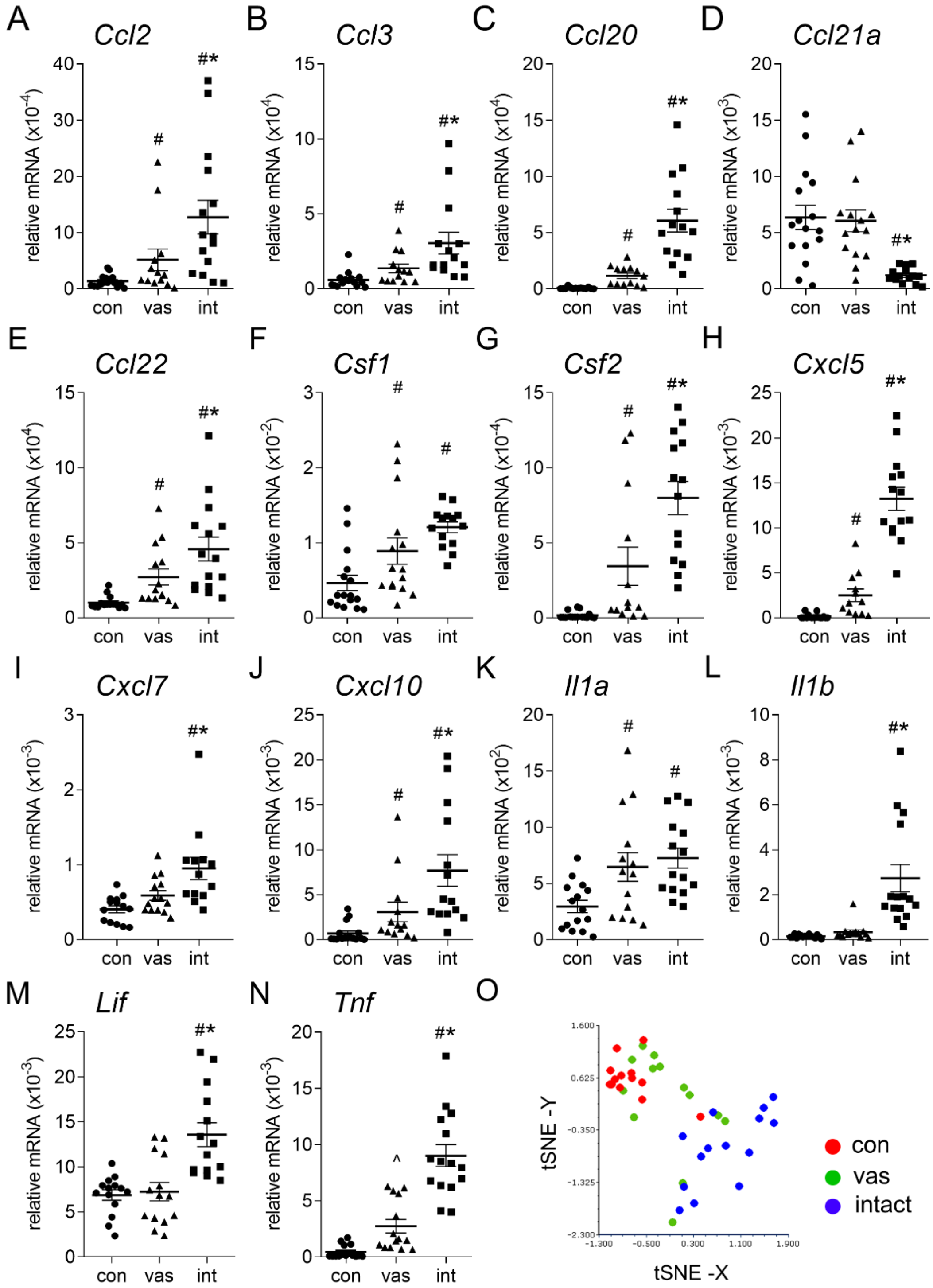

Supplementary Figure 1. Effect of mating with intact males, compared to vasectomized males, on cytokine expression in the uterus. Uterine endometrial tissue was collected from females in estrus (unmated control, con), or 8 h after mating with either intact (int) or vasectomized (vas) males. RNA was extracted, transcribed into cDNA, and expression of *Ccl2* (*A*), *Ccl3* (*B*), *Ccl20* (*C*), *Ccl21a* (*D*), *Ccl22* (*E*), *Csf1* (*F*), *Csf2* (*G*), *Cxcl5* (*H*), *Cxcl7* (*I*), *Cxcl10* (*J*), *Il1a* (*K*), *Il1b* (*L*), *Lif* (*M*), and *Tnf* (*O*) was quantified by qPCR using the delta C(t) method with *Actb* as reference gene. Symbols depict data from individual mice (n = 13-16/group) and mean ± SEM values are shown. Data was analyzed by one-way ANOVA with post-hoc Sidak test, or non-parametric Kruskal Wallis test with post-hoc Dunn's multiple comparisons test. #*P* < 0.05 compared to unmated estrus control group, *P < 0.05 compared to vasectomized-mated group. The tSNE algorithm was applied to concatenated files containing qPCR data for all cytokine transcripts from endometrial tissue of each mouse (*O*) using FSC Express (version 6, DeNovo Software, Los Angeles, CA). Symbols depict data from individual mice, including control estrus mice (red), and females mated to vasectomized males (green) and intact males (blue), n=13-16/group.

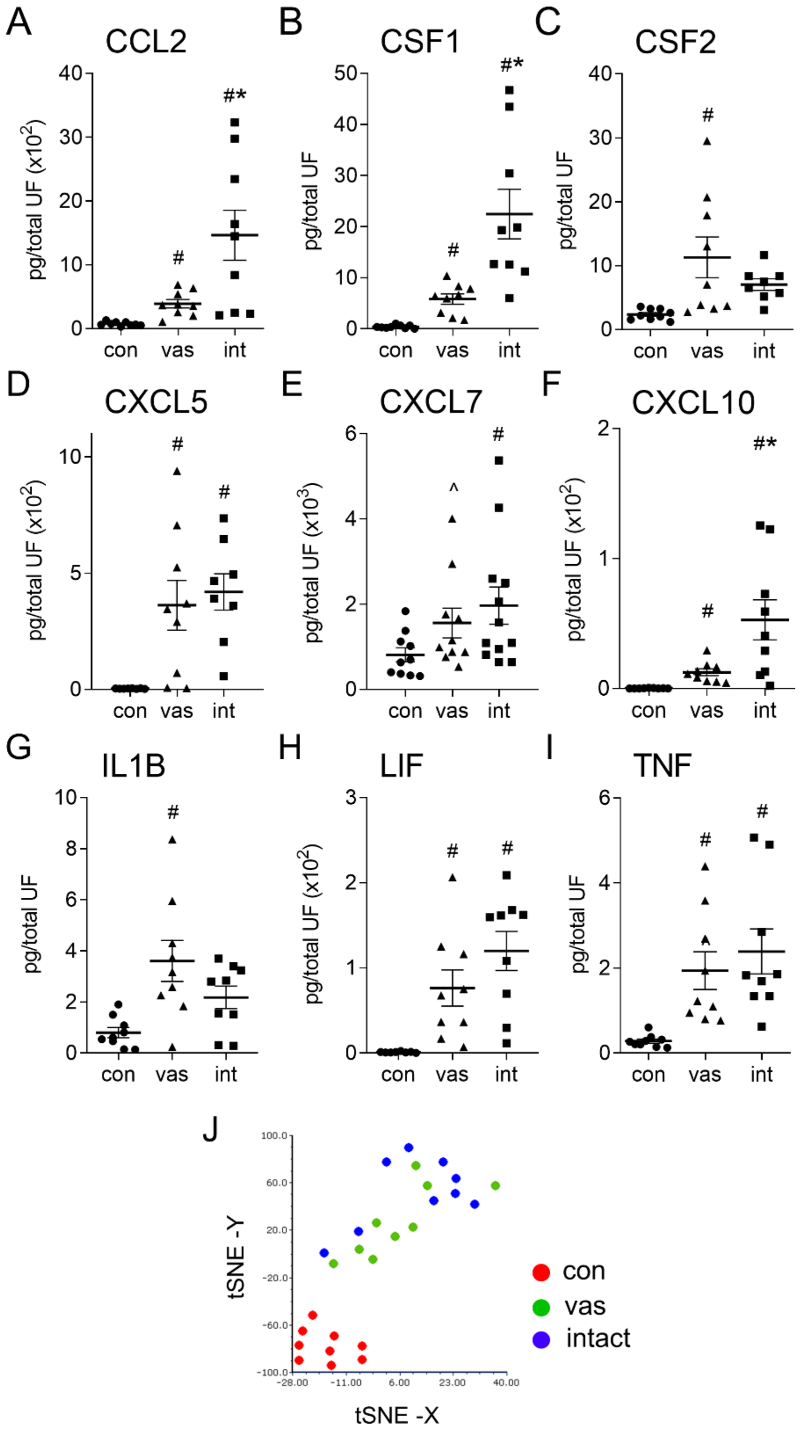

Supplementary Figure 2. Cytokine and chemokine content of uterine luminal fluid after mating with intact males, compared to vasectomized males. Uterine luminal fluid was collected from females in estrus (unmated control, con), or 8 h after mating with either intact (int) or vasectomized (vas) males. Cytokines and chemokines CCL2 (*A*), CSF1 (*B*), CSF2 (*C*), CXCL5 (*D*), CXCL7 (*E*), CXCL10 (*F*), IL1B (*G*), LIF (*H*), and TNF (*I*) were quantified by multiplex microbead assay or ELISA. Symbols depict data from individual mice (n = 8-9/group) and mean ± SEM values are shown. Data was analyzed by one-way ANOVA with post-hoc Sidak test, or non-parametric Kruskal Wallis test with post-hoc Dunn's multiple comparisons test. #$P < 0.05$ compared to unmated estrus control group, *$P < 0.05$ compared to vasectomized-mated group. The tSNE algorithm was applied to concatenated files containing the luminal fluid protein data for all cytokine proteins from each mouse (*J*) using FSC Express (version 6, DeNovo Software, Los Angeles, CA). Each symbol indicates an individual mouse, including control estrus mice (red), and females mated to vasectomized males (green) and intact males (blue), n=9/group.

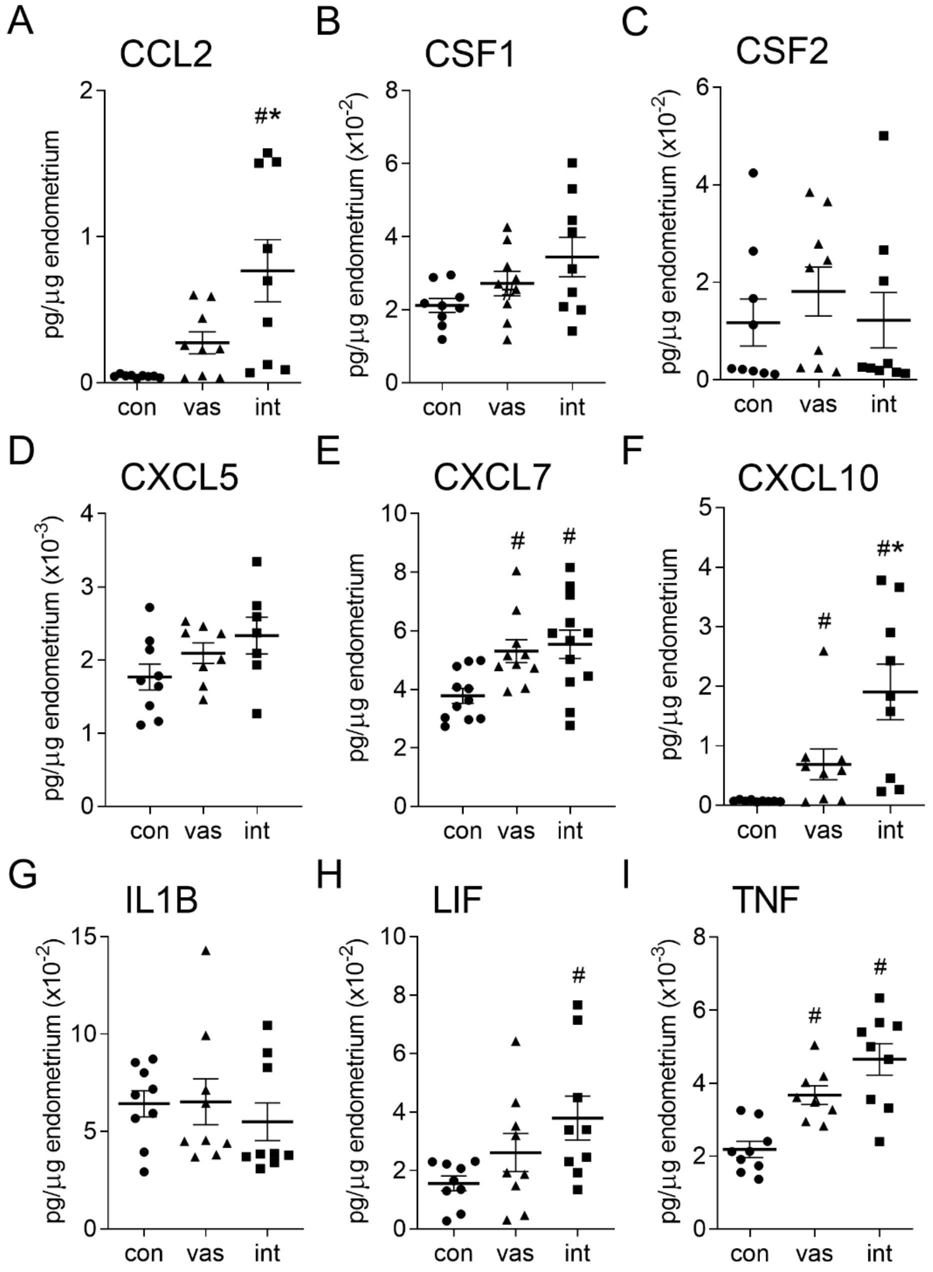

Supplementary Figure 3. Effect of mating with intact males, compared to vasectomized males, on cytokine and chemokine content of uterine endometrial tissue. Uterine endometrial tissue was collected from females in estrus (unmated control, con), or 8 h after mating with either intact (int) or vasectomized (vas) males. Cytokines and chemokines CCL2 (*A*), CSF1 (*B*), CSF2 (*C*), CXCL5 (*D*), CXCL7 (*E*), CXCL10 (*F*), IL1B (*G*), LIF (*H*), and TNF (*I*) were quantified by multiplex microbead assay or ELISA in tissue homogenates. Symbols depict data from individual mice (n = 8-12/group) and mean ± SEM values are shown. Data was analyzed by one-way ANOVA with post-hoc Sidak test, or non-parametric Kruskal Wallis test with post-hoc Dunn's multiple comparisons test. #$P < 0.05$ compared to unmated estrus control group; *$P < 0.05$ compared to vasectomized-mated group.

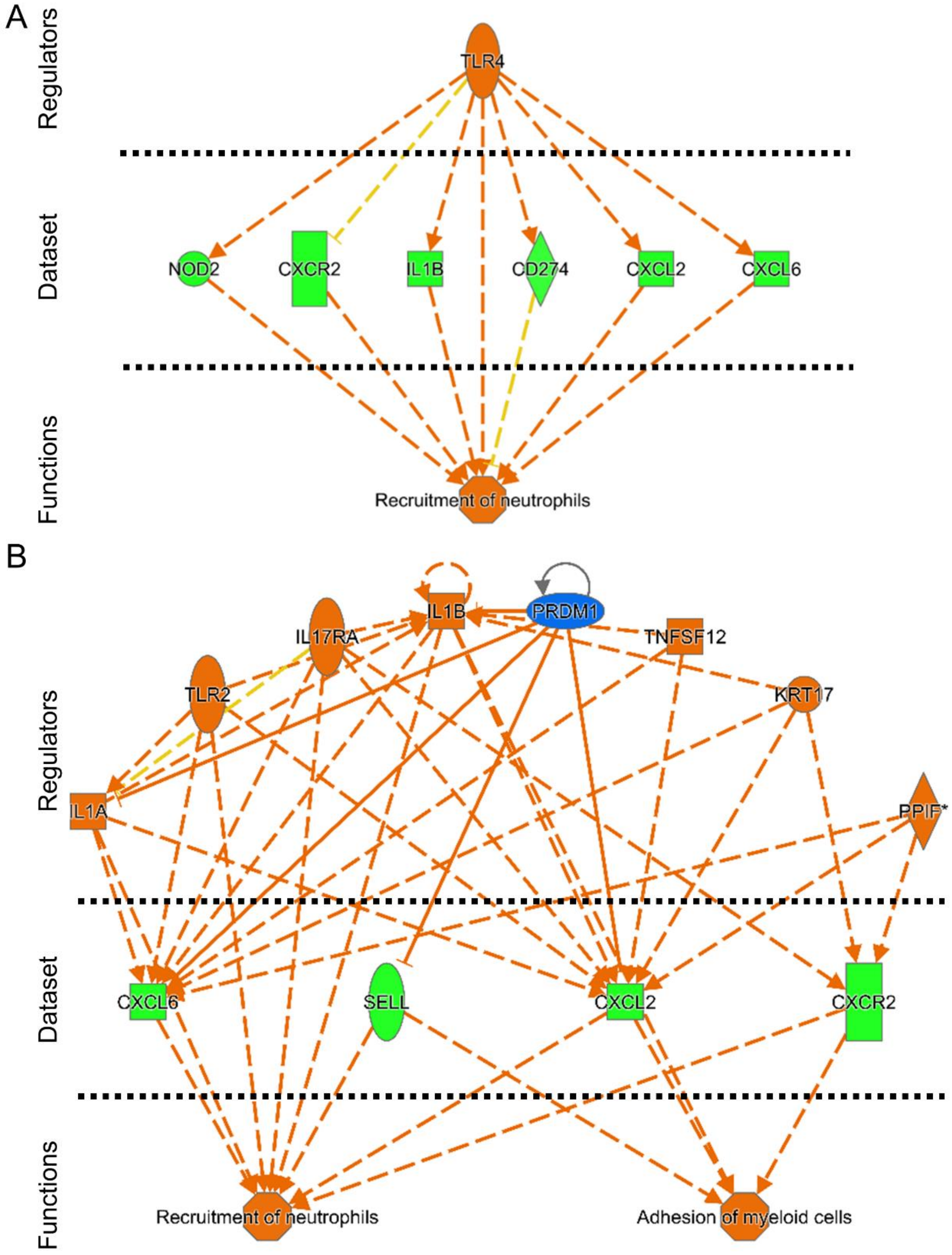

Supplementary Figure 4. Pathway analysis of differentially expressed genes identifies neutrophil recruitment. Microarray data was analyzed by Partek Genomics Suite and Ingenuity Pathway Analysis software. The regulator effects function was used to overlay predicted upstream regulators (regulators), with genes identified as differentially regulated in the dataset (dataset) to predict key functions of the differentially regulated genes (functions). Regulators predicted to be activated (orange) or inhibited (blue) are shown to interact with induced differentially regulated genes (green) in the dataset, leading to the activation (orange) or downstream cellular functions. Connecting lines indicate whether evidence in the literature is consistent with changes observed in the dataset. Connecting lines indicate several predicted relationships that lead to activation (orange), and one relationship inconsistent with the literature (yellow). Cellular functions associated with differentially regulated genes included ‘recruitment of neutrophils’ and ‘adhesion of myeloid cells’ (A, B).

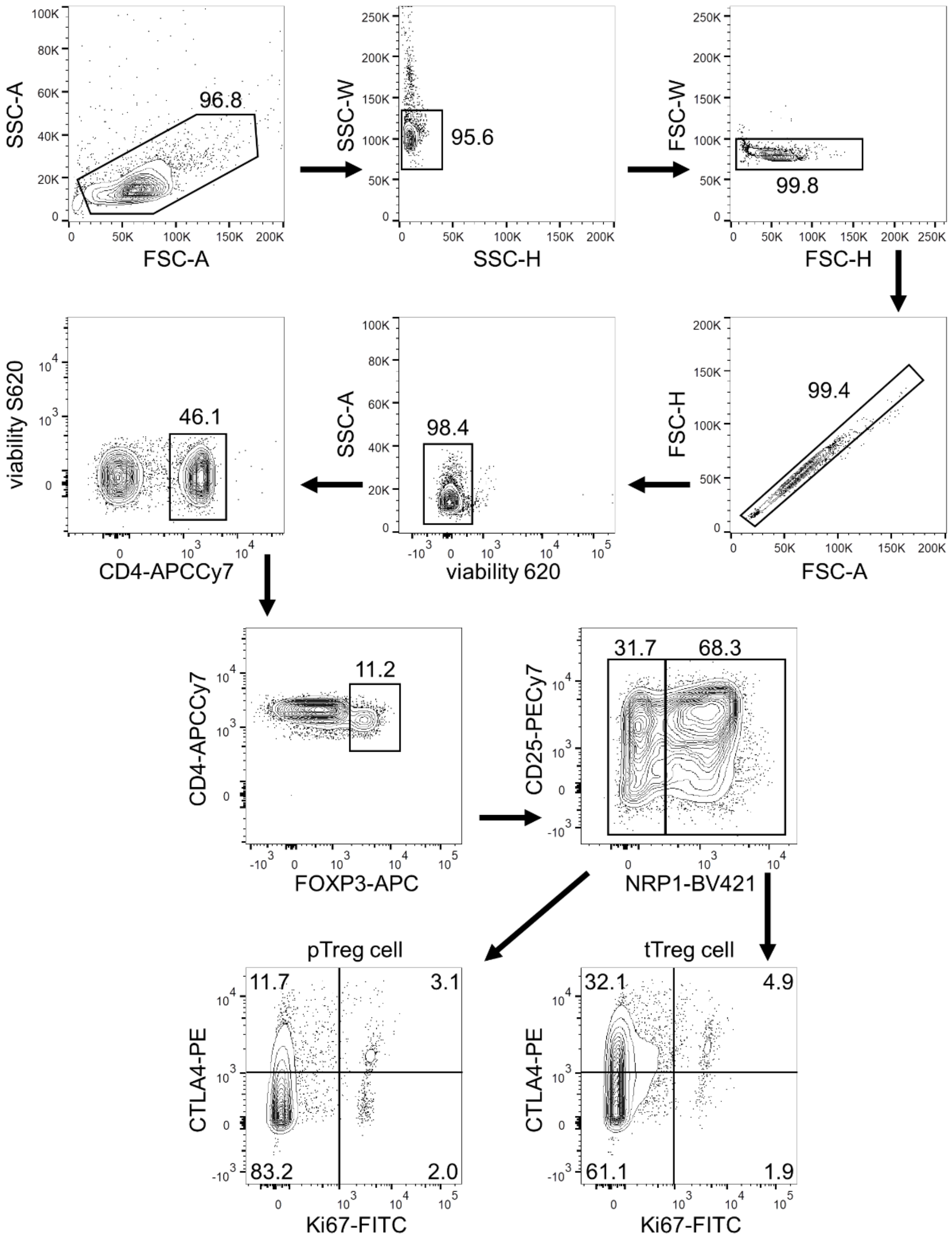

Supplementary Figure 5. Flow cytometry gating strategy. Uterus-draining para-aortic lymph nodes were recovered from mice and $CD4^+$ T cells were analyzed by flow cytometry. Gates were established to include viable lymphocytes and exclude debris and doublets using forward and side scatter profiles and viability dye exclusion. Lymphocytes positive for CD4 were gated. For the T cell population, cells positive for FOXP3 within the CD4 cell gate were defined as Treg cells. Gates were established within the $CD4^+FOXP3^+$ Treg cell population to distinguish $NRP1^-$ (pTreg) and $NRP1^+$ (tTreg) cells. CD25, Ki67 and CTLA4 were measured within total Treg, tTreg and pTreg populations. Numbers in quadrants are % of population.

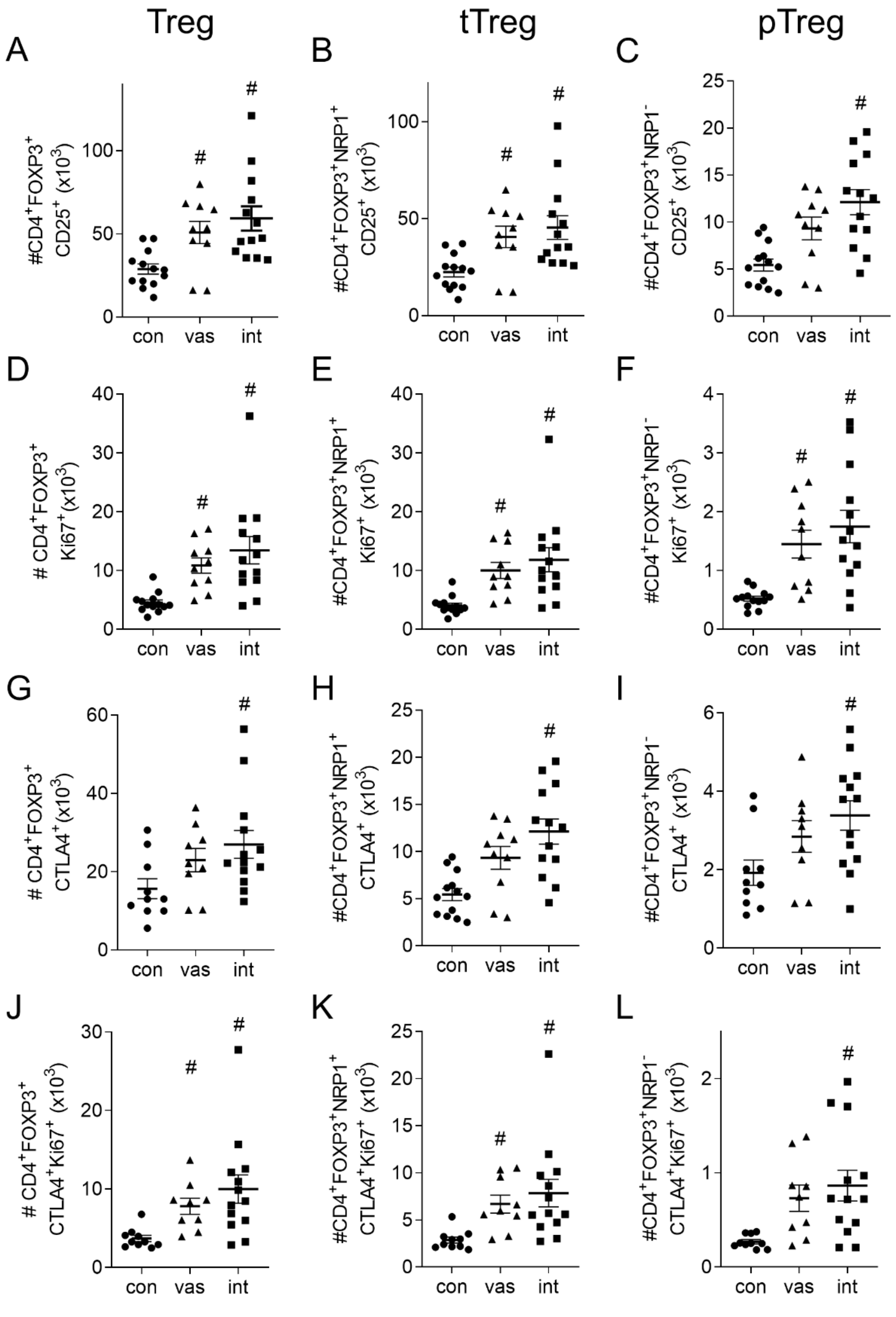

Supplementary Figure 6. Effect of mating with intact males, compared with vasectomized males, on regulatory T cells in lymph nodes draining the uterus. Uterus-draining para-aortic lymph nodes (PALN) were collected from female mice in estrus (unmated control, con), or on day 3.5 pc after mating with either intact (int) or vasectomized (vas) males. T cells were analyzed by flow cytometry for CD4, FOXP3, CD25, NRP1, Ki67, and CTLA4 expression. Numbers of $CD4^+FOXP3^+$ Treg, $CD4^+FOXP3^+NRP1^{-/lo}$ pTreg cells and $CD4^+FOXP3^+NRP1^+$ tTreg cells expressing CD25 (*A*-*C*), Ki67 (*D*-*F*), CTLA4 (*G*-*I*), or both Ki67 and CTLA4 (*J*-*L*), were quantified. Symbols depict data from individual mice (n = 10-13/group) and mean ± SEM values are shown. Data was analyzed by one-way ANOVA with post-hoc Sidak test, or non-parametric Kruskal Wallis test with post-hoc Dunn's multiple comparisons test. #*P* < 0.05 compared to unmated estrus control group, **P* < 0.05 compared to vasectomized-mated group.

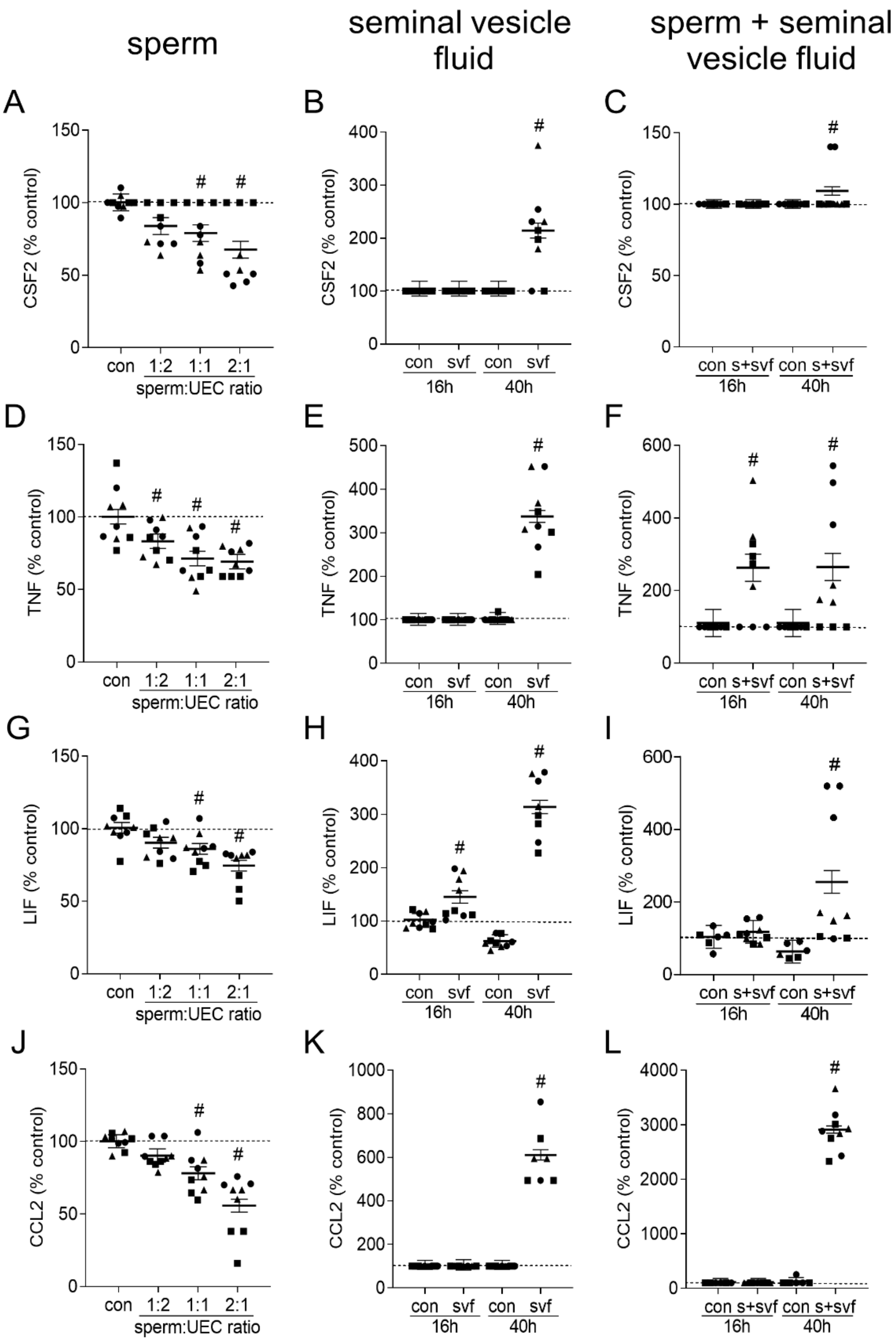

Supplementary Figure 7. **Effect of sperm and seminal plasma on cytokine release from uterine epithelial cells *in vitro*.** Uterine epithelial cells (UEC) were isolated from estrus CBAF1 mice and incubated with control media alone (con), sperm (1:2, 1:1, 2:1 sperm:UEC ratio) (*A, D, G, J*), seminal vesicle fluid (svf; 5%) (*B, E, H, K*), or a combination of 2:1 sperm:UEC + 5% svf (*C, F, I, L*), for 16 hr. Culture media was replaced and collected after a further 24 h culture period, then CSF2 (*A-C*), TNF (*D-F*), LIF (*G-I*), and CCL2 (*J-L*) were quantified by multiplex microbead assay in 16 h (*A-L)* and 40 h supernatants (*B, C, E, F, H, I, K, L*). Mean baseline cytokine concentration in control supernatants was ~200 pg/10^5 cells/24 h (CSF2); ~40 pg/10^5 cells/24 h (TNF), ~90 pg/10^5 cells/24 h (LIF), and ~50 ng/10^5 cells/24 h (CCL2). Symbols depict data from 3 experiments (with 3 replicates/experiment), and estimated marginal mean ± SEM values are shown. Data was analyzed by mixed model ANOVA, with experiment as covariate. # P < 0.05 compared to medium alone control.

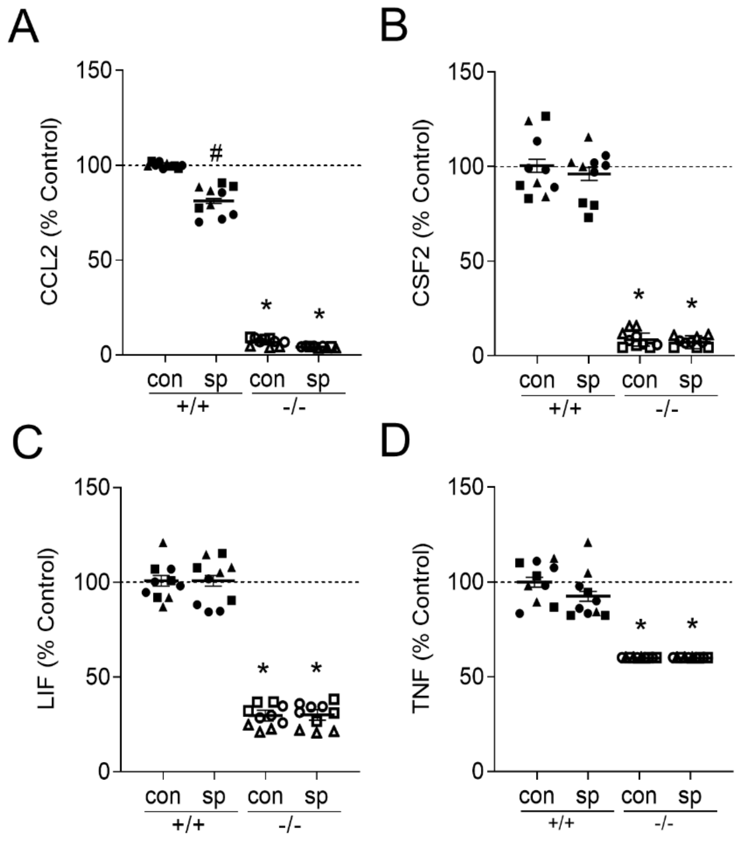

Supplementary Figure 8. Effect of TLR4 deficiency on production of proinflammatory cytokines by uterine epithelial cells in vitro. Uterine epithelial cells (UEC) were isolated from estrus *Tlr4*$^{+/+}$ or *Tlr4*$^{-/-}$ mice and incubated with control media alone (con), or sperm (2:1 sperm:UEC ratio) (*A*-*D*), for 16 h. Supernatants were collected then CCL2 (*A*), CSF2 (*B*), LIF (*C*), and TNF (*D*) were quantified by multiplex microbead assay. See legend to Supplementary Figure 7 for mean baseline cytokine concentration in control supernatants. Symbols depict data from 3 experiments (with 3 replicates/experiment), and estimated marginal mean ± SEM values are shown. Data was analyzed by mixed model ANOVA, with experiment as covariate. # $P < 0.05$ compared to control media alone within genotype, * $P < 0.05$ compared to *Tlr4*$^{+/+}$ cells with same treatment.

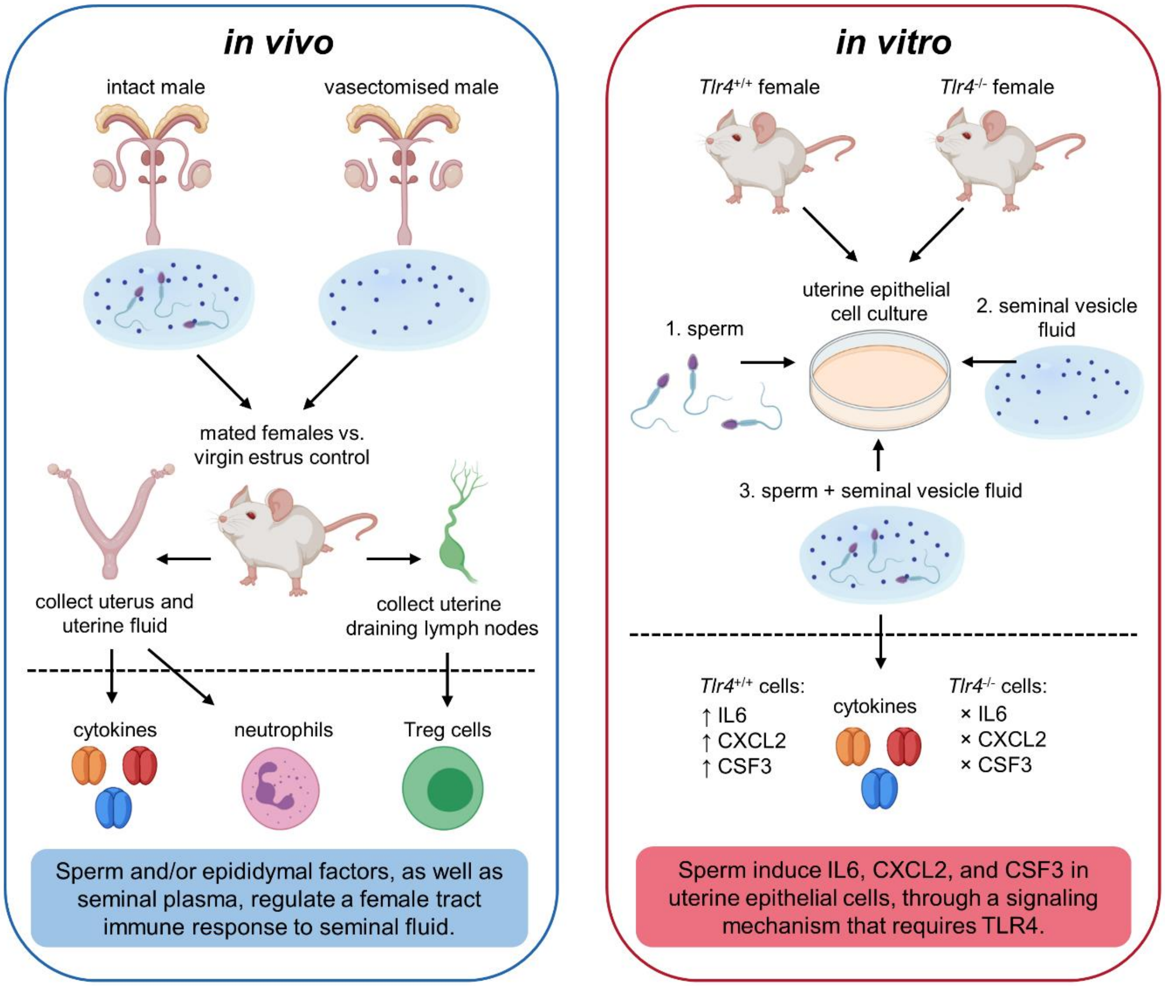

Supplementary Figure 9. A graphical abstract summarizing the experiments and outcomes reported in this study. Figure created with BioRender.com.

Supplemental Table 1. Canonical signaling pathways with that are either significantly (*P* value < 5.0E-02) activated (Z-score >2) or inhibited (Z-score <-2) in the endometrium of vasectomized mated females compared to virgin estrus control females.

Activated

Ingenuity Canonical Pathways	*P* value	Z-score
Acute Phase Response Signaling	1.20E-06	3.16
Cardiac Hypertrophy Signaling (Enhanced)	9.33E-05	2.83
HMGB1 Signaling	1.70E-04	2.65
Role of PRR in Recognition of Bacteria and Viruses	3.72E-04	2.45
IL6 Signaling	5.75E-04	2.12
Cholecystokinin/Gastrin-mediated Signaling	7.41E-04	2.83
LPS-stimulated MAPK Signaling	1.35E-03	2.65
Production of Nitric Oxide and ROS in Macrophages	1.78E-03	2.53
Dendritic Cell Maturation	2.95E-03	2.12
iNOS Signaling	6.76E-03	2
ErbB Signaling	1.00E-02	2.45
UVC-Induced MAPK Signaling	1.20E-02	2
NF-κB Signaling	1.35E-02	2.12
UVB-Induced MAPK Signaling	2.82E-02	2
RANK Signaling in Osteoclasts	3.16E-02	2.24
IL17A Signaling in Airway Cells	4.17E-02	2

Supplemental Table 2. Bioinformatics prediction of novel seminal fluid signaling mediators. Ingenuity Pathway analysis was used to predict upstream regulators of the differentially expressed genes between the intact (int) and vasectomized (vas) mated groups. Upstream regulators with a significant *P* value (< 5.0E-2) and predicted as activated (Z-score > 2) or inhibited (Z-score <-2) are listed, together with the number of associated differentially expressed (DE) targets within the dataset.

Activated

Upstream Regulator	Z-score	*P* value	# DE targets
MYD88	2.33	3.61E-07	10
IL17RA	2.18	4.68E-07	5
TLR4	2.03	5.61E-07	11
KRT17	2.24	6.73E-07	5
CD38	2.8	2.08E-06	8
TNFSF12	2.41	4.88E-06	6
SCAP	2.24	6.10E-06	6
IL5	2.43	1.19E-04	6
IFNG	2.22	1.53E-04	11
PPIF	2.24	2.04E-04	5
RELA	2.08	3.87E-04	5
MAP3K8	2.19	1.17E-03	5
TLR3	2.19	1.83E-03	5
IL1B	2.19	1.89E-03	5

Inhibited

Upstream Regulator	Z-score	P-value	# DE targets
INSIG1	-3	2.73E-09	9
PPARA	-2.16	2.43E-04	9
POR	-2.43	5.59E-04	6

Supplemental Table 3. PCR primers used to quantify mRNAs.

Target	Forward primer	Reverse primer	[Primer]	Accession #
bAct	5'- TGTGATGGTGGGTATGGGTC -3'	5'- ACACGCAGCTCATTGTA -3'	0.25μm	NM_007393
Ccl2	5'- CCAGCAAGATGATCCCAATGA -3'	5'- TCTCTTGAGCTTGGTGACAAAAAC -3'	0.25μM	NM_011333
Ccl3	5'- TGCCCTTGCTGTTCTTCTCTG -3'	5'- AACGATGAATTGGCGTGGA -3'	0.25μM	NM_011337
Ccl5	5'- TGCCAACCCAGAGAAGAAGTG -3'	5'- TTACTGAGTGGCATCCCCAAG -3'	0.5μm	NM_013653
Ccl20	5'- TCACAAGACAGATGGCCGATG -3'	5'- CACAGCCCTTTTCACCCAGTT -3'	0.5μm	NM_016960
Ccl22	5'- TTCAGACTTCCTTGGCCTCCA -3'	5'- TGCTGCAGACCAAGAAACAGG -3'	0.25μM	NM_009137
Ccl21	5'- TCCAACTCACAGGCAAAGAGG -3'	5'- GCAGATGTGATGGTTGAAGCA -3'	0.25μm	NM_011124
Csf1	5'- GTGACCATCCGTCCGAATGTT -3'	5'-TGGACAATCAAAGGCTGAGGC -3'	0.25μm	NM_007778
Csf2	5'– CCTGGGCATTGTGGTCTACAG -3'	5'- GGCATGTCATCCAGGAGGTT -3'	0.05μm	NM_009969
Csf3	5'- GCAGACACAGTGCCTAAGCCA -3'	5'- CATCCAGCTGAAGCAAGTCCA -3'	0.25μm	NM_009971
Cxcl1	5'- ATTGTATGGTCAACACGCACG -3'	5'- TTTGAACGTCTCTGTCCCGAG -3'	0.5μm	NM_008176
Cxcl2	5'- GAACTGCGCTGTCAATGCCT -3'	5'- CCGCCCTTGAGAGTGGCTAT -3'	0.5μm	NM_009140
Cxcl5	5'- TTCCATCTCGCCATTCATGC -3'	5'- CGGTTAAGCAAACACAACGCA -3'	0.25μm	NM_009141
Cxcl7	5'- GCCTGCCCACTTCATAACCTC -3'	5'- CGCAGTTCGATATATGGGTCC -3'	0.5μm	NM_023785
Cxcl10	5'- TCCATCACTCCCCTTTAGCCA -3'	5'- TGTCTCAGGACCATGGCTTGA -3'	0.25μm	NM_021274
IL1α	5'- CCGACCTCATTTTCTTCTGG -3'	5'- GTGCACCCGACTTTGTTCTT -3'	0.1μm	NM_010554.4
IL1β	5'- CCCAAGCAATACCCAAAGAA -3'	5'- GCTTGTGCTCTGCTTGTGAG -3'	0.5μm	NM_008361.3
IL6	5'- ACAACCACGGCCTTCCCTAC -3'	5'- TCCACGATTTCCCAGAGAACA -3'	1μm	NM_031168
Lif	5'- CGCCAATGCTCTCTTCATTTC -3'	5'- TCCGATGCTCCACCAACT -3'	0.5μm	NM_008501
Tnf	5'- GTAGCCCACGTCGTAGCAAAC -3'	5'- CTGGCACCACTAGTTGGTTGTC -3'	1μm	NM_013693
