# Supplementary material for: Sperm modulate uterine immune parameters relevant to embryo implantation and reproductive success in mice
Source: Commun Biol. 2021 May 14;4:572. doi: 10.1038/s42003-021-02038-9 (PMC8121928; doi:10.1038/s42003-021-02038-9)
Supplement: Supplementary file 2 — Description of Additional Supplementary Files [file 42003_2021_2038_MOESM2_ESM.pdf]

Description of Additional Supplementary Files

File name: Supplementary Data 1

Description: Genes differentially regulated in microarray data with fold change >1.5 and FDR < 5.0E-02. Intact vs unmated virgin estrus control comparison. FC = Fold-Change; FDR = False Discovery rate P Value.

File name: Supplementary Data 2

Description: Genes differentially regulated in microarray data with fold change >1.5 and FDR < 5.0E-02. Vasectomized vs unmated virgin estrus control comparison. FC = fold-change; FDR = false discovery rate P value.

File name: Supplementary Data 3

Description: Canonical signalling pathways with that are either significantly (P value < 5.0E-02) activated (Z-score >2) or inhibited (Z-score <-2) in the endometrium of intact mated females compared to virgin estrus control females.

File name: Supplementary Data 4

Description: Genes differentially regulated in microarray data with fold change >1.5 and FDR < 5.0E-02. Intact vs Vasectomized comparison. FC = fold-change; FDR = false discovery rate P value.

File name: Supplementary Data 5

Description: Bioinformatics prediction of novel seminal fluid signaling mediators. Ingenuity Pathway analysis was utilized to predict upstream regulators of the differentially expressed genes between the intact (int) mated and unmated virgin estrus (con) females. Upstream regulators with a significant P value (< 5.0E-02) and predicted as activated (Z-score > 2) or inhibited (Z-score <-2) are listed, together with the number of associated differentially expressed (DE) targets within the dataset.

File name: Supplementary Data 6

Description: Bioinformatics prediction of novel seminal fluid signaling mediators. Ingenuity Pathway Analysis was utilized to predict upstream regulators of the differentially expressed genes between the vasectomized (vas) mated and unmated virgin estrus (con) females. Upstream regulators with a significant P value (< 5.0E-02) and predicted as activated (Z-score > 2) or inhibited (Z-score < -2) are listed, together with the number of associated differentially expressed (DE) targets within the

dataset.Figure_1.xlsx. Normalized absorption spectra of different OCP variants in 0.8 M phosphate, presented in Figure 1. The tabs are labeled according to the panels in the figure.
