# Supplementary material for: Sperm modulate uterine immune parameters relevant to embryo implantation and reproductive success in mice
Source: Commun Biol. 2021 May 14;4:572. doi: 10.1038/s42003-021-02038-9 (PMC8121928; doi:10.1038/s42003-021-02038-9)
Supplement: Supplementary file 9 — Reporting Summary [file 42003_2021_2038_MOESM9_ESM.pdf]

Corresponding author(s): Sarah A Robertson

Last updated by author(s): Mar 10, 2021

Reporting Summary

Nature Research wishes to improve the reproducibility of the work that we publish. This form provides structure for consistency and transparency in reporting. For further information on Nature Research policies, see our Editorial Policies and the Editorial Policy Checklist.

Statistics

For all statistical analyses, confirm that the following items are present in the figure legend, table legend, main text, or Methods section.

n/a	Confirmed	
☐	☒	The exact sample size (*n*) for each experimental group/condition, given as a discrete number and unit of measurement
☐	☒	A statement on whether measurements were taken from distinct samples or whether the same sample was measured repeatedly
☐	☒	The statistical test(s) used AND whether they are one- or two-sided<br>*Only common tests should be described solely by name; describe more complex techniques in the Methods section.*
☐	☒	A description of all covariates tested
☐	☒	A description of any assumptions or corrections, such as tests of normality and adjustment for multiple comparisons
☐	☒	A full description of the statistical parameters including central tendency (e.g. means) or other basic estimates (e.g. regression coefficient) AND variation (e.g. standard deviation) or associated estimates of uncertainty (e.g. confidence intervals)
☒	☐	For null hypothesis testing, the test statistic (e.g. *F*, *t*, *r*) with confidence intervals, effect sizes, degrees of freedom and *P* value noted<br>*Give P values as exact values whenever suitable.*
☒	☐	For Bayesian analysis, information on the choice of priors and Markov chain Monte Carlo settings
☒	☐	For hierarchical and complex designs, identification of the appropriate level for tests and full reporting of outcomes
☒	☐	Estimates of effect sizes (e.g. Cohen's *d*, Pearson's *r*), indicating how they were calculated

Our web collection on statistics for biologists contains articles on many of the points above.

Software and code

Policy information about availability of computer code

Data collection	N/A
Data analysis	Statistical analysis was conducted using Partek Genomics Suite (Partek Inc, Chesterfield MO); Ingenuity Pathway Analysis, version 47547484 (Qiagen, Hilden, Germany); SPSS for Windows, version 20.0 software (SPSS Inc, Chicago IL); GraphPad Prism, version 9.0.0 (GraphPad Software LLC, San Diego CA) and FCS Express v6.06 (De Novo Software, California, USA).

For manuscripts utilizing custom algorithms or software that are central to the research but not yet described in published literature, software must be made available to editors and reviewers. We strongly encourage code deposition in a community repository (e.g. GitHub). See the Nature Research guidelines for submitting code & software for further information.

Data

Policy information about availability of data

All manuscripts must include a data availability statement. This statement should provide the following information, where applicable:

- Accession codes, unique identifiers, or web links for publicly available datasets
- A list of figures that have associated raw data
- A description of any restrictions on data availability

The microarray data in this manuscript is deposited in the National Centre for Biotechnology Information Gene Expression Omnibus and are accessible through GEO series accession number GSE167485. The authors declare that all other data supporting the findings of this study are available within the paper and its supplementary information files.

Field-specific reporting

Please select the one below that is the best fit for your research. If you are not sure, read the appropriate sections before making your selection.

- [x] Life sciences
- [ ] Behavioural & social sciences
- [ ] Ecological, evolutionary & environmental sciences

For a reference copy of the document with all sections, see nature.com/documents/nr-reporting-summary-flat.pdf

Life sciences study design

All studies must disclose on these points even when the disclosure is negative.

Sample size	Sample size for in vivo experiments was determined as the minimum number of mice required to achieve statistical significance between treatment groups as determined by power analysis calculation, assuming treatments alter mean values for gene expression, cytokine concentration, or cell numbers by ≥30% (alpha = 0.05, beta = 0.2, power = 0.8).<br>Sample size for in vitro experiments was determined as the minimum number of replicates required to achieve statistical significance between treatment groups as determined by power analysis calculation, assuming treatments alter mean values for ene expression, cytokine concentration, or cell numbers by ≥40% (alpha = 0.05, beta = 0.2, power = 0.8).
Data exclusions	For microarray experiments and in vitro cell culture experiments, no data was removed from analysis. For in vivo experiments (qPCR, cytokine protein, flow cytometry, and immunohistochemistry, outliers were identified by ROUT test (Q = 1) in GraphPad Prism, and removed from analysis.
Replication	Microarray experiments utilized 4 biological replicates per experimental group, each comprised of tissues from 4 mice, and several differentially expressed genes were confirmed by PCR. Three replicates of in vitro experiments were undertaken and all showed similar results. For in vivo experiments, ethical principles for use of animals in research prohibited replication beyond the minimum number of mice required for statistical significance.
Randomization	Allocation of mice to experimental groups was random in all experiments.
Blinding	Investigators were not blinded to experimental groups. Blinding was not possible in the current experiments where the same individual investigators applied treatments and measured outcomes.

Reporting for specific materials, systems and methods

We require information from authors about some types of materials, experimental systems and methods used in many studies. Here, indicate whether each material, system or method listed is relevant to your study. If you are not sure if a list item applies to your research, read the appropriate section before selecting a response.

Materials & experimental systems

n/a	Involved in the study	
[ ]	[x]	Antibodies
[x]	[ ]	Eukaryotic cell lines
[x]	[ ]	Palaeontology and archaeology
[ ]	[x]	Animals and other organisms
[x]	[ ]	Human research participants
[x]	[ ]	Clinical data
[x]	[ ]	Dual use research of concern

Methods

n/a	Involved in the study	
[x]	[ ]	ChIP-seq
[ ]	[x]	Flow cytometry
[x]	[ ]	MRI-based neuroimaging

Antibodies

Antibodies used	CD16/CD32 purified, cat# 553142, lot# 7081871, clone 2.4G2, labelling concentration 1 µg/mL, BD Biosciences<br>CD4-APC-Cy7, cat# 552051, lot# 7055747, clone GK1.5, labelling concentration 2 µg/mL, BD Biosciences<br>CD25-PE-Cy7, cat#552880 lot# 7172888, clone PC61, labelling concentration 2 µg/mL, BD Biosciences<br>NRP1-BV421, cat# 145209, lot# B246181, clone 3E12, labelling concentration 0.3 µg/mL, BioLegend<br>FOXP3-APC, cat# 17-5773-82, lot# 4330883, clone FJK-16s, labelling concentration 2 µg/mL, Thermo Fisher Scientific<br>Ki67-FITC, cat# 11-5698-82, clone SolA15, labelling concentration 4 µg/mL, Thermo Fisher Scientific<br>CTLA4-PE, cat# 553720, lot# 7082872, clone UC10-4F10-11, labelling concentration 2 µg/mL, BD Biosciences<br>Lot numbers are given where known.
Validation	The concentration of each antibody used in flow cytometry experiments was determined by labeling mouse lymphocytes in a serial dilution ranging from 0.1 µg/mL to 5 µg/mL per million cells. The optimal concentration was selected as the lowest amount of antibody required to clearly define cell populations without causing excessive spread or compensation issues.<br>Technical data sheets from manufacturer's show validation data and are listed below;

CD16/CD32 https://www.bdbiosciences.com/ds/pm/tds/553142.pdf
CD4 https://www.bdbiosciences.com/ds/pm/tds/552051.pdf
CD25 https://www.bdbiosciences.com/ds/pm/tds/552880.pdf
CTLA4 https://www.bdbiosciences.com/ds/pm/tds/553720.pdf
NRP1 https://www.biolegend.com/en-us/global-elements/pdf-popup/brilliant-violet-421-anti-mouse-cd304-neuropilin-1-antibody-8731?filename=Brilliant%20Violet%20421trade%20anti-mouse%20CD304%20Neuropilin-1%20Antibody.pdf&pdfgen=true
FOXP3 https://www.thermofisher.com/order/genome-database/dataSheetPdf?producttype=antibody&productsubtype=antibody_primary&productId=17-5773-82&version=133
Ki67 https://www.thermofisher.com/order/genome-database/dataSheetPdf?producttype=antibody&productsubtype=antibody_primary&productId=11-5698-82&version=133

Animals and other organisms

Policy information about studies involving animals; ARRIVE guidelines recommended for reporting animal research

Laboratory animals	Experiments utilized laboratory mice (mus musculus) of various inbred and genetic strains. Mice were pathogen-free CBA x C57Bl/6 F1 (CBAF1) and BALB/c females, and BALB/c and C57Bl/6 males, obtained from Laboratory Animal Services, University of Adelaide (Adelaide, Australia), or the Animal Resource Centre (Perth, Australia). Mice with a null mutation in the Tlr4 gene (Tlr4−/− mice, on a BALB/c background) were sourced from Prof. Akira (Osaka University, Osaka, Japan) and supplied by Prof. Paul Foster (University of Newcastle, Newcastle, Australia). All mice were co-housed in specific pathogen free conditions at the University of Adelaide Medical School Animal House on a 12 h light–dark cycle and were given standard rodent chow (Envigo, Teklad Diets, Madison WI) and water ad libitum. Experimental females were 9-16 wks and males were 12-28 wks of age at the time of use.
Wild animals	The study did not involve wild animals.
Field-collected samples	The study did not involve field-collected samples.
Ethics oversight	Animal usage was in accordance with the Australian Code of Practice for the Care and Use of Experimental Animals, and experiments were approved by the University of Adelaide Animal Ethics Committee (approval numbers: M-2010-095, M-2014-023, M-2017-006).

Note that full information on the approval of the study protocol must also be provided in the manuscript.

Flow Cytometry

Plots

Confirm that:

- [x] The axis labels state the marker and fluorochrome used (e.g. CD4-FITC).
- [x] The axis scales are clearly visible. Include numbers along axes only for bottom left plot of group (a 'group' is an analysis of identical markers).
- [x] All plots are contour plots with outliers or pseudocolor plots.
- [x] A numerical value for number of cells or percentage (with statistics) is provided.

Methodology

Sample preparation	Uterine draining para-aortic lymph nodes (PALN) were collected from CBAF1 females in estrus, or on day 3.5 pc following mating with BALB/c males. Single cell suspensions of PALN were prepared by gentle crushing between two frosted glass slides and cells were washed in complete RPMI media (Thermo Fisher Scientific; RPMI + 10% FCS + 2% penicillin/streptomycin). Cells were counted on a haemocytometer and 1,000,000 cells were plated in a 96-well U bottom plate (Corning, New York), washed twice in PBS then incubated with fixable viability stain 620 (1:1000 in PBS, BD Biosciences, San Jose, CA) at RT in the dark for 20 min before washing in FACS buffer and incubation with Fc receptor block (as per manufacturer's instructions, BD Biosciences). Cells were then stained with fluorophore-conjugated monoclonal antibodies CD4-APCCy7 (GK1.5, 2 μg/mL; BD Biosciences), CD25-PECy7 (PC61, 2 μg/mL; BD Biosciences), and NRP1-BV421 (3E12, 0.3 μg/mL; BioLegend, San Diego, CA) in FACS buffer for 25 min at 4 degrees C. Cells were fixed and permeabilized using the FOXP3 Staining Buffer Set; Thermo Fisher Scientific) according to manufacturer's instructions. Fixed cells were labelled with FOXP3-APC (FJK-16s, 2 μg/mL; Thermo Fisher Scientific), Ki67-FITC (SolA15, 4 μg/mL; Thermo Fisher Scientific) and CTLA4-PE (UC10-4F10-11, 2 μg/mL; BD Biosciences) diluted in permeabilization wash (FOXP3 Staining Buffer Set; Thermo Fisher Scientific).
Instrument	BD FACS Canto II, model 33862, serial number V96301297
Software	Data was acquired use BD FACS Diva, version 6 software and analysed using FlowJo, version 10.6.1 (both BD Biosciences, San Jose, CA).
Cell population abundance	Cells were not sorted in this study.
Gating strategy	Single lymphocytes were identified and debris and dead cells exclude based on a series of scatter gates, SSC-A vs FSC-A -> SSC-W vs SSC-H -> FSC-W vs FSC-H -> FSC-H vs FSC-A. Cells were then applied to a SSC-A vs fixable viability stain 620, with FVS620 positive cells excluded from the analysis. CD4+ lymphocytes were identified on a FVS620 vs CD4-APCCy7 plot. FOXP3+ Treg cells were then gated on a CD4-APCCy7 vs FOXP3-APC plot. NRP1- pTreg and NRP1+ tTreg cells were gated on a CD25-PECy7 vs NRP1-BV421 plot. Each Treg subset was then placed on a CTLA4-PE vs Ki67-FITC plot to assess expression of these markers within each population using a quadrant gate. All fluorescent marker gates where determined using unstained,

isotype and/or fluorescent minus one controls.

☒ Tick this box to confirm that a figure exemplifying the gating strategy is provided in the Supplementary Information.
